# Influence of climatic variation on microbial communities during organic Pinot noir wine production

**DOI:** 10.1371/journal.pone.0296859

**Published:** 2024-02-28

**Authors:** Aghogho Ohwofasa, Manpreet Dhami, Junwen Zhang, Bin Tian, Christopher Winefield, Stephen L. W. On

**Affiliations:** 1 Department of Wine, Food and Molecular Biosciences, Lincoln University, Lincoln, New Zealand; 2 Centre of Foods for Future Consumers, Lincoln University, Lincoln, New Zealand; 3 Manaaki Whenua—Landcare Research, Lincoln, New Zealand; Universidade do Minho, PORTUGAL

## Abstract

To assess the possible impact of climatic variation on microbial community composition in organic winemaking, we employed a metabarcoding approach to scrutinize the microbiome in a commercial, organic, Pinot noir wine production system that utilizes autochthonous fermentation. We assessed microbial composition across two vintages (2018 and 2021) using biological replicates co-located at the same winery. Microbial dynamics were monitored over four important fermentation time points and correlated with contemporaneous climate data. Bacterial (*R*_ANOSIM_ = 0.4743, p = 0.0001) and fungal (*R*_ANOSIM_ = 0.4738, p = 0.0001) compositions were different in both vintages. For bacteria, *Lactococcus* dominated the diversity associated with the 2018 vintage, while *Tatumella* dominated the 2021 vintage. For fungal populations, while *Saccharomyces* were abundant in both vintages, key differences included *Starmerella*, copious in the 2018 vintage; and *Metschnikowia*, substantive in the 2021 vintage. Ordination plots correlated the climatic variables with microbial population differences, indicating temperature as a particularly important influence; humidity values also differed significantly between these vintages. Our data illustrates how climatic conditions may influence microbial diversity during winemaking, and further highlights the effect climate change could have on wine production.

## Introduction

Globally, and in New Zealand specifically, climate change poses an immense risk to the wine industry [1]. In comparison to soil and cultivar, climate has a greater impact on fruit composition and berry development [2]. Climatic conditions change from year to year in every wine-producing region. The “vintage effect” comes from such changes resulting in differences in quality, output, and typicity from one year to another [3]. Macro-climatic variables such as precipitation and temperature do have direct and indirect consequences on agricultural productivity [4, 5]. These consequences arise due to their impact on key viticultural characteristics like grape maturation and harvest dates [5]. Viticultural practices can also affect fruit quality and acidity, microbiota, sugar content, pH and potassium concentrations which impact wine making decisions [5–7]. Viticultural practices are already requiring emendations to adapt to climate change [8].

The increase in the use of next-generation sequencing (NGS) for analyzing the microbiome of wine and other fermented products is highlighted by its application by many research groups [9–13], indicating that the high-throughput sequencing approach is an efficient tool for characterizing microbial communities [14]. In New Zealand, this approach has been utilized in reporting key microbial information from wine-growing regions around the country [15]. Nevertheless, not all wine-making regions, or production methods, have been explored.

The Waipara area in the South Island of New Zealand (43°02’49.0"S 172°47’12.4"E), is characterized by its cool, dry and warm temperate climate [16]. The Canterbury plains are usually swept by cool marine winds, however, due to coastal hills protecting the region, it is said to be viticulturally warmer than the plains to the south [17]. The unique regional properties of Pinot noir wine from this region have since been described [18]. Furthermore, a recent oenological study in the region [19] applied MALDI-TOF in characterizing cultured yeast strains and for the first time reported the presence of *Candida californica* in a New Zealand vineyard. A later study demonstrated how environmental conditions had more impact on the bacterial populations as compared to the fungal, over the course of a complete alcoholic fermentation period [20]. However, each of these studies analyzed only a single vintage and thus, we are currently unable to tell if such changes to the microbial community are consistent over two or more vintages. Internationally, related studies such as Steenwerth, Morelan [21] for example, examined large American viticultural areas (AVA’s) over two vintages. One of their conclusions was the fact that the differential abundance of some genera did change with growing season precipitation and vintage. Such investigations highlight the value (and need) for similar projects investigating other regions, especially where spontaneous fermentations are relied upon.

The aim of our study was to use an NGS approach in analyzing the diversity of bacteria and fungi across two vintages using key time points (start of fermentation, total soluble solids (TSS) dropped by 6–8˚ Brix, TSS dropped by half, and end of fermentation). With this, we tested whether (a) bacterial community and (b) fungal community dynamics and composition are conserved across two vintages. To clearly uncover differences in wine fermentation microbiome that can be attributed to the climate associated with a given vintage, two co-located environments in each vintage were studied, thus acting as biological replicates. Furthermore, we aimed to correlate the microbial information with the climatic data from both vintages, to test the hypothesis that, even within a sub region, climatic differences across vintages would have an influence on microbes relevant to wine making. Thus, here we compared the microbial diversity of two vintages using NGS approaches and correlated this with climatic data, in ferments conducted in the same winery in the Waipara region of New Zealand.

## Materials and methods

### Sample collection, metabarcoding and sequencing

Pinot noir grapes were harvested over two vintages from the same block of vineyard that consistently uses an organic approach. Samples from the 2018 vintage were harvested on March 22, 2018 (21.7°Brix). For the 2021 samples, the relevant timepoints using the corresponding brix value were selected from our previous study [20]. Thus, DNA Extraction, metabarcoding and sequencing followed the same protocol as used previously Ohwofasa, Dhami [20]. In brief, fermentation was undertaken using the same winemaking practices (including spontaneous fermentation and cap management) consistently employed by the winemaker across all vintages. Grapes consisted of 80% destemmed bunches, and 20% whole clusters. For each vintage, grapes were split into two batches with each batch undergoing an autochthonous fermentation in large tanks (1500 L) placed in two locations, representing biological replicates. Note that both fermenters were treated the same in terms of plunging and coverings. For plunging, this was done manually once per day. Additionally, no SO₂ was added to the musts. The locations are the winery—an indoor environment (replicate A) and the vineyard–an outdoor environment (replicate B). It is important to state that a lid was always used to cover both tanks to keep out insects or any other contaminants. The vineyard (outdoors) fermentation was stationed about 1 KM from the winery (indoors). If the weather forecast predicts rainfall or if precipitation starts, a hardcover was placed on the fermenter (vineyard). This was necessary to ensure rainwater is completely excluded from the process. All time points, locations and the dates of sampling are shown in Table 1.

**Table 1:** Sampling dates and the different fermentation stages adopted.

	S1 (Start of fermentation– 21°Brix)	S2 (TSS dropped by 6–8˚Brix)	S3 (TSS dropped by half)	S4 (End of fermentation–less than 1˚ Brix)
**Winery (2021)**	21SW1 (March 18, 2021)	21SW2 (March 19, 2021)	21SW3 (March 20, 2021)	21SW4 (April 4, 2021)
**Vineyard (2021)**	21SV1 (March 17, 2021)	21SV2 (March 18, 2021)	21SV3 (March 19, 2021)	21SV4 (April 3, 2021)
**Winery (2018)**	18SW1 (March 27, 2018)	18SW2 (March 28, 2018)	18SW3 (March 29, 2018)	18SW4 (April 12, 2018)
**Vineyard (2018)**	18SV1 (March 26, 2018)	18SV2 (March 26, 2018)	18SV3 (March 27, 2018)	18SV4 (April 11, 2018)

For further analysis, the samples collected as depicted above were immediately preserved at -80 ˚C. The method outlined by Wei, Wu [22] was then used to generate the pellet. Briefly, upon defrosting at 4 ˚C, the grape juice was thoroughly shaken to mix evenly and 5ml of each sample was collected in sterile 15ml tubes. A short (ca. 60 seconds) vortex mixing was done, followed by centrifugation at 3000 g for 5 minutes at 4 ˚C (Heraeus Multifuge X3R, Thermo Scientific). The pellet generated was washed three times with 5 ml of UltraPure distilled water (Invitrogen) and stored at -20 ˚C for DNA extraction.

DNA extraction utilized the soil protocol of the Mag-Bind Environmental DNA 96 kit (OMEGA) albeit with slight modification. Modification involved the physical lysing of cells where we employed TissueLyser II (QIAGEN) for 3 minutes at 20 Hz. The 1.5% agarose gel electrophoresis was applied to assess the quality of the extracted DNA. Using a DeNovix DS-11 spectrophotometer, we estimated the total DNA concentration.

We adapted the two-step amplification process as described by San Juan, Castro [23] for metabarcoding. The first step involves PCR amplification of the V4 region of the 16S ribosomal RNA gene for bacteria using 515F/806R primer pairs [23]. For fungi analysis, using an equimolar mix of primer pairs (LSU200 (A)-F/LSU481(A)-R and LSU200-F/LSU481-R [24], we targeted the large subunit (LSU) ribosomal DNA region. The KAPA 3G PCR plant kit (Omega Bio-tek) was used for first step PCR using the manufacturer’s recommended protocols (scaled down to 15μl). The following PCR conditions were utilized: Initial denaturation at 95°C for 120 s, 35 cycles of denaturation at 95°C for 20 s, 52.5°C (Bacteria)/55°C (fungi) for 20 s, and 72°C for 30 s. A final extension was completed at 72°C for 10 minutes.

The template for the second step PCR were the products yielded from the first step PCR. Barcoded primers were used here and conditions applied to the PCR were as follows; Initial denaturation at 95°C for 2 min, 5 cycles of 95°C for 20 s (denaturation), 50°C for 20 s (annealing), 72°C for 30 s (extension), and final extension at 72°C for 2 min. SeraMag Magnetic Speed-Beads [25] were adopted to purify the second-stage PCR products generated. This is required to normalize concentration and remove primer dimers. Using Qubit (dsDNA HS Assay Kit, Invitrogen, Carlsbad, United States), we determined the DNA concentration. Thereafter, we pooled all libraries in an equimolar manner using amplicon length and the number of samples found in each library. LabChip GX Touch Nucleic Acid Analyzer (PerkinElmer, Waltham, United States) was applied to assess DNA concentration and the quality of the final pooled library. Lastly, using a nano-format, sequencing was done with the Illumina MiSeq platform (phiX spike 10%, 250 × 2 cycles, NanoSeq kit) at Auckland Genomics Facility (University of Auckland).

We utilized the DADA2 (version 1.20.0) pipeline [26] for the analysis of the obtained raw sequences. Before using DADA2, sequence reads downloaded from Illumina were converted from BCL to fastq format. This was achieved using the bcl2fastq2 (version 2.20) Illumina software. Claident (version 2018.05.08) [27] was thereafter needed to demultiplex all the raw sequences. Sequences at this point are ready for DADA2 pipeline and this performed quality filtering, merging, chimera removal, and finally inferred Amplicon Sequence Variants (ASVs). The ASVs inferred were then used for taxonomic assignment. For prokaryotes, assignment was done using the SILVA v132 16S rRNA database [28]. While the UNITE fungal taxonomic reference [29] was utilized for eukaryotic organisms. To obtain a finer taxonomic resolution, relevant ASVs were probed using the Basic local alignment search tool (BLAST) [30]. The New Zealand eScience Infrastructure (NeSI) high performance computing (HPC) environment was applied for bioinformatics analysis.

### Historical climate data

Weather reports associated with the Waipara region for the 2018 and 2021 growing season (including the harvest and fermentation period) were sourced from Harvest [31] (Table 2). Averaged values for the climatic factors served as environmental variables used in fitting our model (S1 Table). Additionally, to clearly identify climatic factors that were statistically different in both vintages, we applied a Paired Samples Wilcoxon Test [32] in R (v4.1.0) due to the large variance in some climatic variables e.g Rain (S1 Fig).

**Table 2:** Weather report for Waipara region. (Accessed: July, 2023).

Period	Min. Temp. (˚c)	Average Temp. (˚c)	Max. Temp. (˚c)	Min. Relative Humidity (%)	Average Relative Humidity (%)	Max. Relative Humidity (%)	Rain (mm)
**October 2017**	2.5	12.3	25.1	33	79	100	92.4
**November 2017**	2.6	14.2	25.2	27	74	100	1
**December 2017**	5.2	18.4	33.6	18	67	99	38.4
**January 2018**	6.6	19.1	34.6	30	81	100	144
**February 2018**	4.3	17.2	32.4	24	72	99	185.4
**March 2018**	4.6	16.1	28.9	32	76	99	9.2
**April 2018**	-1.1	11.5	27.1	29	75	99	116.2
**Average ± SD**	**3.5 ± 2.49**	**15.5 ± 2.96**	**29.6 ± 3.98**	**27.6 ± 5.19**	**74.9 ± 4.6**	**99.4 ± 0.53**	**83.8 ± 70.18**
**October 2020**	-0.3	12.8	26.8	25	70	95	51
**November 2020**	3.2	14	28.2	29	73	94	60.6
**December 2020**	3.4	15.7	30.9	21	68	96	110.2
**January 2021**	5.6	17.1	36.6	21	70	93	38
**February 2021**	1.3	16.6	32.8	18	71	96	10
**March 2021**	1.8	16.3	31.2	10	67	94	29.8
**April 2021**	-1.5	13.8	28.3	31	66	94	21.4
**Average ± SD**	**1.9 ± 2.39**	**15.2 ± 1.64**	**30.68 ± 3.33**	**22.14 ± 7.08**	**69.3 ± 2.43**	**94.6 ± 1.13**	**45.8 ± 33.13**

### Statistical analysis

For analysis of microbial diversity, the R programming language (v4.1.0) was utilized. Phyloseq package [33] and other packages used are listed in S File 1 and 2. We created one phyloseq object each for bacterial and fungal data. We then transformed the ASV abundances to relative abundance to account for dissimilarities in library sizes. Transformed data were utilized for further analysis. Taxa found in 80% of the samples with an abundance ≥ 0.0001 were retrieved as the core microbiome associated with each vintage and their replicates.

To determine suitable tests to be employed, we performed the Shapiro-Wilk normality tests [34]. Differences within each vintage were shown using the Shannon diversity index. Thereafter, data were explored using Bray-curtis distance matrix ordination via a non–metric multidimensional scaling plot (NMDS). Furthermore, using our weather data, we correlated our variables with the generated NMDS plot through the “envfit” function in vegan [35]. For improved visualization, “ordispider” (vegan) was used to highlight all samples to the centroid of its vintage. The “adonis” function (vegan) was also applied to test group level significance via PERMANOVA [36]. Finally, to establish ASVs that were significantly different, DESeq2 was utilized [37]. By applying the Analysis of similarities (ANOSIM) with 999 permutations [38], we tested the null hypothesis by comparing the within and between group similarities at the community level.

## Results

### Climate data overview

Relative humidity (average and maximum) was shown to be significantly different (p < 0.05) and higher in the 2018 vintage (S1E and S1F Fig). Although rain as a climatic variable showed no significant difference, a greater range was observed in the 2018 vintage (Table 2 and S1G Fig) i.e 1mm in November 2017 versus 185mm in February 2018—the highest in a two-year period (2018–2020) [31]. Temperature records also showed that on average, a higher maximum temperature was associated with the year 2020 while temperature minima and means was greater in 2017 (S2 Table).

### The core bacterial community and composition of vintage 2018 showed high diversity when compared to the 2021 vintage

The 179 ASVs generated was used to assign taxonomy and this gave rise to a total of 110 genera identified across the 2018 and 2021 vintages: 37 of those genera were present in both vintages while 17 appeared only in the 2021 vintage. Specifically, genera found in the 2021 vintage include *Tatumella* (52.48%), *Bacillus* (14.13%), *Gluconobacter* (6.95%), *Fructobacillus* (5.32%), *Sphingomonas* (4.64%), and *Hyphomicrobium* (2.56%). Genera such as *Lactococcus*, *Bradyrhizobium*, and members of the Caulobacteraceae family were also found but in minor quantities of less than 2% each (S3 Table).

The 2018 vintage which was more diverse had 56 genera which were absent in the 2021 vintage. Thus, none was overly dominant as the case of *Tatumella* in the 2021 vintage. Genera found here include *Lactococcus* (24.68%), *Gluconobacter* (11.37%), *Bacillus* (10.29%), *Sphingomonas* (6.54%), Orbaceae family (5.47%), *Lactobacillus* (5.11%), *Acetobacter* (4.80%), *Komagataeibacter* (4.72%), *Hyphomicrobium* (3.91%), *Bradyrhizobium* (2.18%). The presence of other genera was very limited; for example, *Leuconostoc*, *Tatumella*, *Enhydrobacter*, *Klebsiella*, and *Pseudomonas* were found to have an abundance of less than 2% (S3 Table).

**Fig 1:**
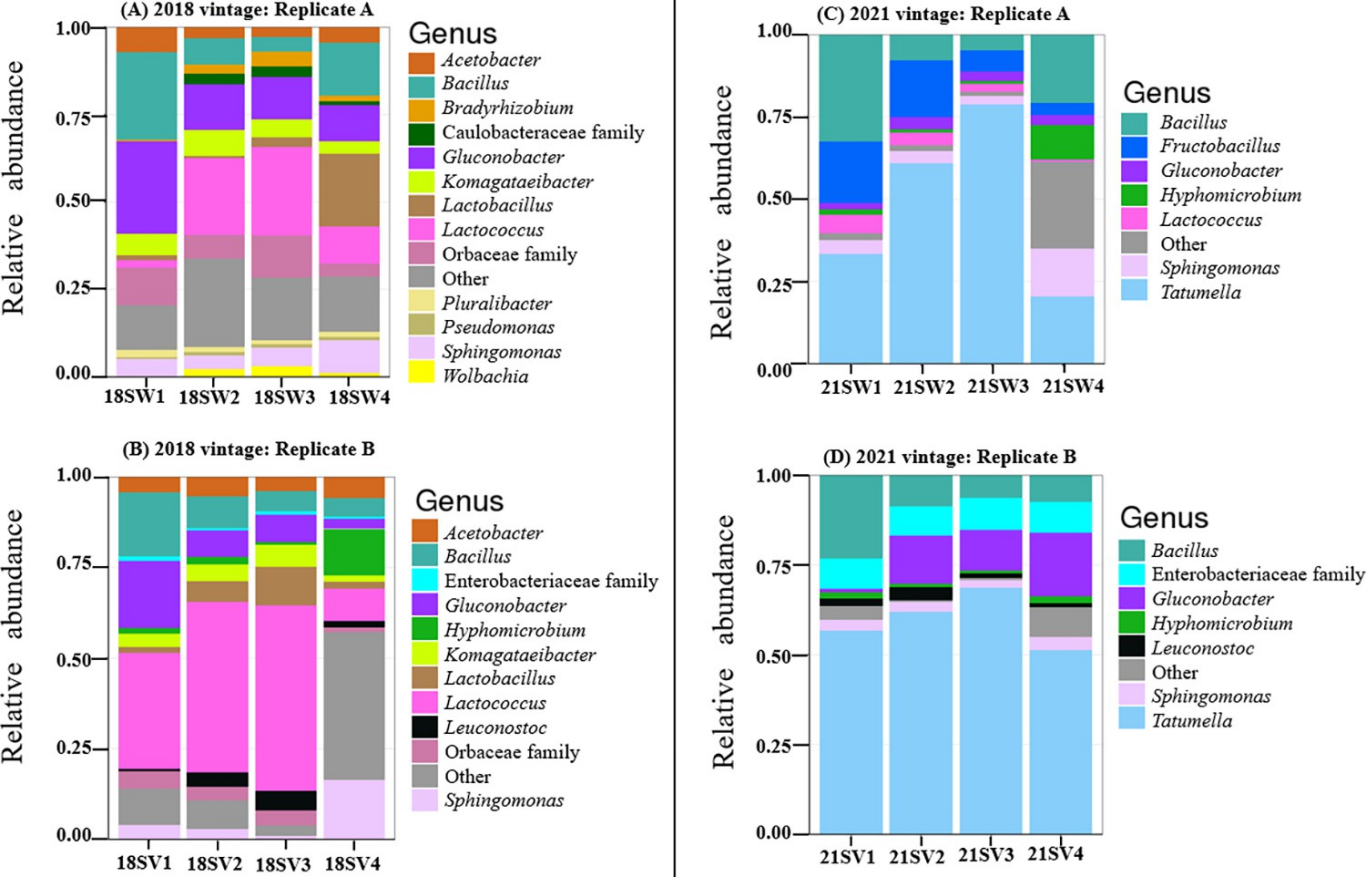
Relative abundance of bacterial genera in (A) 2018 vintage replicate A; (B) 2018 vintage replicate B; (C) 2021 vintage replicate A; (D) 2021 vintage replicate B. Relative abundances are shown at the genus level. Bacteria which could not be resolved to the genus level are shown at the family level. Similarities between the replicates in each vintage can be observed. SW = Samples Winery; SV = Samples Vineyard.

Significant differences (*R*_ANOSIM_ = 0.4743, *p* = 0.0001) at the community level were observed when bacterial communities associated with the vintages were compared. Thus, relative abundance of the bacterial community varied significantly across both vintages. Fig 1 shows the relative abundance plots.

**Fig 2:**
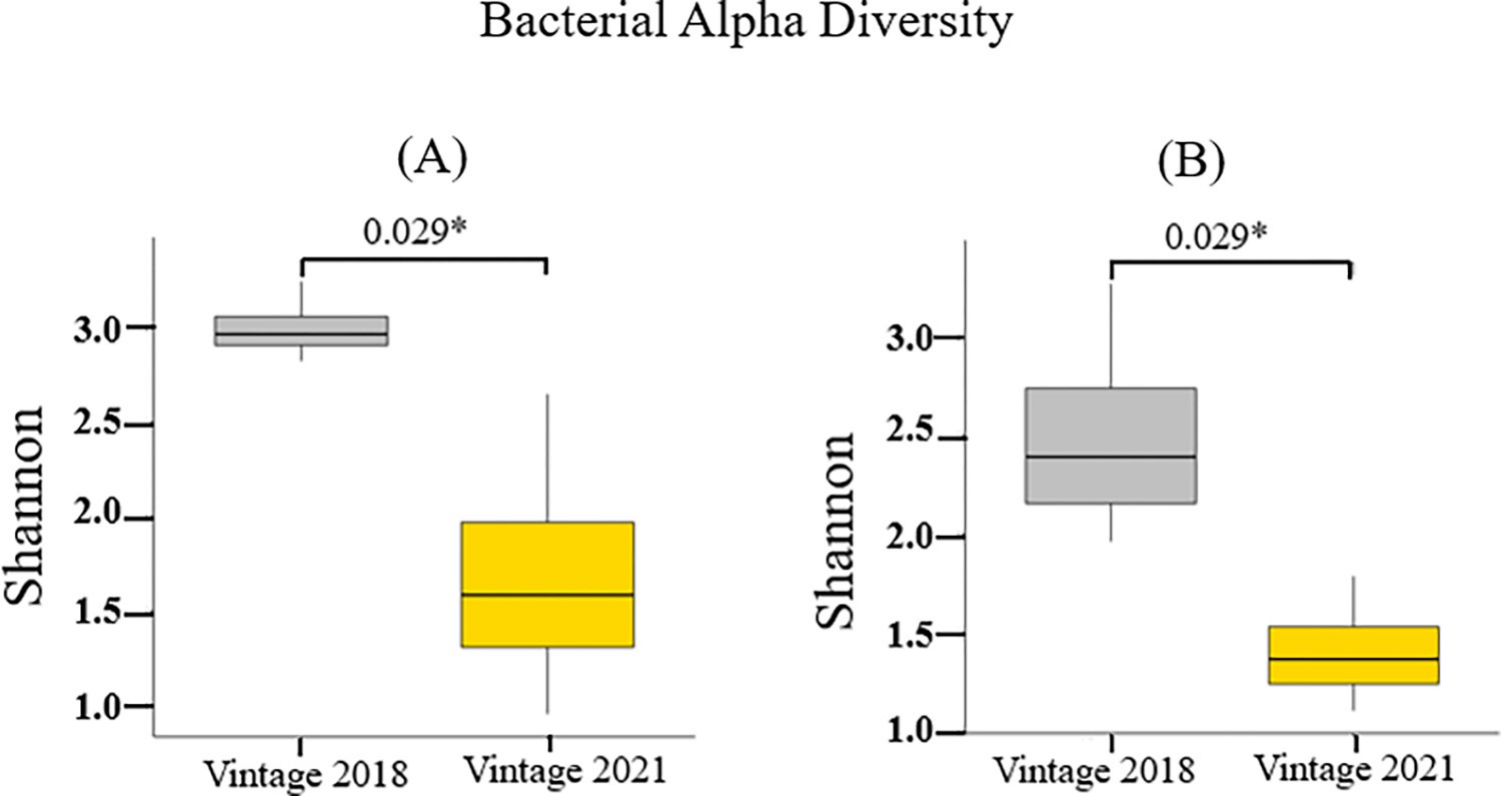
Bacterial alpha diversity (Shannon diversity index) of the ferment samples from (A) Replicate A; (B) Replicate B. The 2018 vintage displayed a higher diversity compared to the 2021 vintage. A similar conclusion can be drawn from both replicates.

The above was supported by Shannon diversity index as a measure of Alpha diversity. Fig 2 shows that this was higher in the 2018 vintage (p = 0.029). This indicates that in comparison with the 2021 vintage, the bacterial community associated with the 2018 vintage had a higher bacterial diversity (Fig 2). Similarly, using PERMANOVA to examine if vintage had an impact on the bacterial community composition gave rise to significant findings (PERMANOVA: F = 17.08, R^2^ = 0.55, p = 0.0001) (S3A Fig). In terms of differential abundance, from the analysis of DESeq2 output, eight bacterial genera were significantly different when both vintages are compared. Specifically, *Fructobacillus*, *Tatumella*, and *Bacillus* were abundant in the 2021 vintage, while other genera such as *Gluconobacter*, *Acetobacter*, *Pluralibacter*, *Komagataeibacter*, and *Lactobacillus* dominated the 2018 vintage (S3 and S4 Tables).

### Core fungal community of vintage 2018 and 2021 were similar but compositional differences such as *Starmerella* (2018 vintage) and *Metschnikowia* (2021 vintage) were seen

The 79 ASVs were distributed among 41 Fungal genera. Fifteen genera were found in both vintages, while thirteen were uniquely associated with each vintage. As expected, *Saccharomyces* made up a large part of this in both the 2021 and the 2018 vintage. In the former, *Saccharomyces* had an abundance of 44.3%. Other genera found here include *Metschnikowia* (25.6%), *Aureobasidium* (16.3%), *Hanseniaspora* (4.84%), and *Cladosporium* (4.76%) (S5 Table).

**Fig 3:**
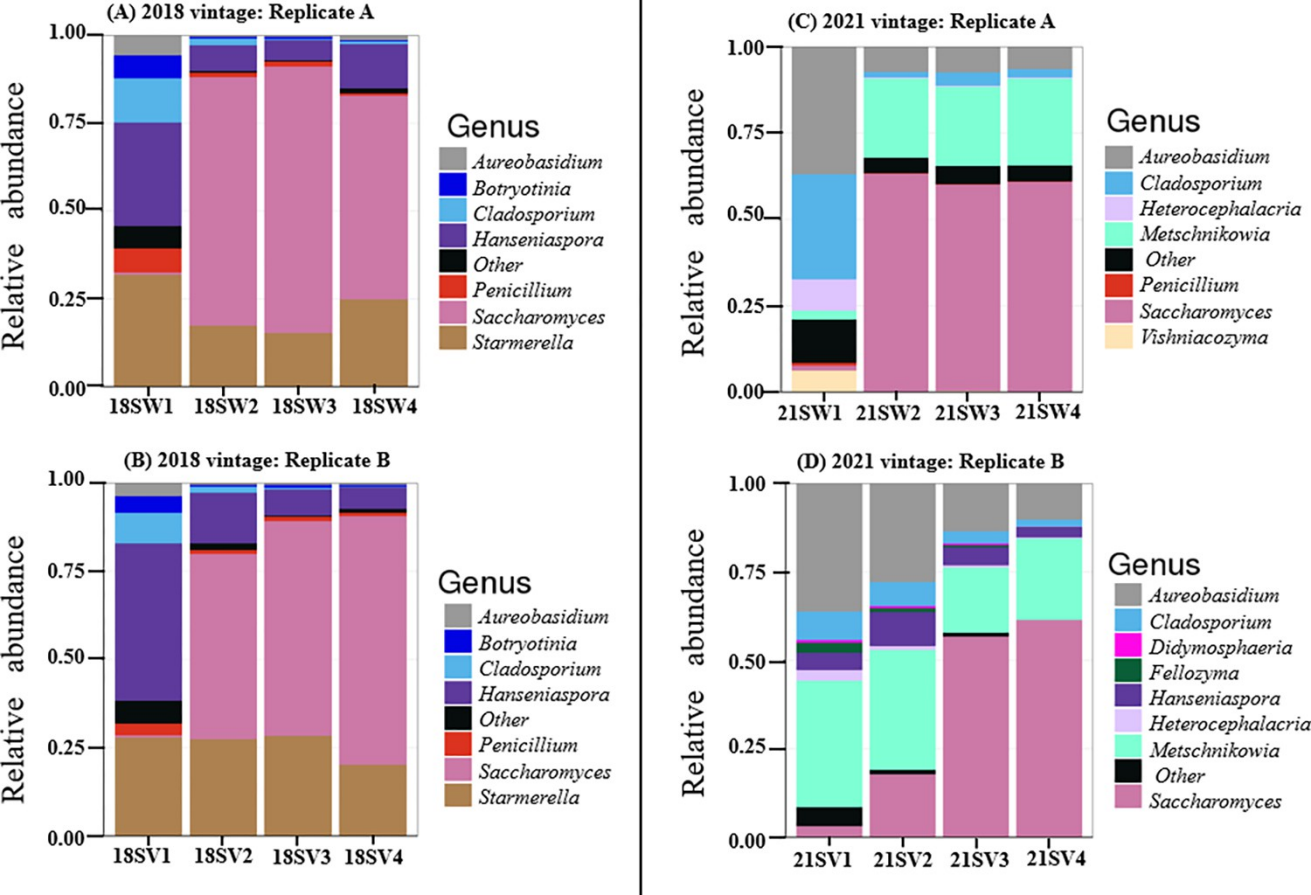
Relative abundance of Fungal genera in (A) 2018 vintage replicate A; (B) 2018 vintage replicate B; (C) 2021 vintage replicate A; (D) 2021 vintage replicate B. Relative abundances are shown at the genus level. Replicates in each vintage bear close resemblance. SW = Samples Winery; SV = Samples Vineyard.

As with the 2021 vintage, *Saccharomyces* made up almost half of the 2018 vintage with an abundance of 49.4%. *Starmerella* (23.8%), *Hanseniaspora* (16.3%), *Cladosporium* (3.0%), and *Aureobasidium* (1.5%) followed this. Genera such as *Penicillium*, *Didymosphaeria*, *Heterocephalacria*, and *Torulaspora* were found in both vintages but in minute abundances of less than 2% each (S5 Table). Fig 3 shows the fungal relative abundance plots.

The Shannon diversity index measuring overall alpha diversity showed no significant difference (p > 0.05; S2 Fig). Fungal communities were similar in both vintages, most likely due to the abundance of *Saccharomyces* as expected with a typical fermentation. However, compositional differences were confirmed (*R*_ANOSIM_ = 0.4738, p = 0.0001). Thus, PERMANOVA reported that the fungal community composition observed in both vintages was significantly influenced by vintage (PERMANOVA: F = 15.277, R^2^ = 0.52, p = 0.0001) (S3B Fig). Analysis of our DESeq2 output revealed that *Starmerella bacillaris*, *Pichia kluyveri*, *Hanseniaspora*, *Torulaspora*, and *Saturnispora* amongst others, were significantly abundant in the 2018 vintage as compared to the 2021 vintage. For the 2021 vintage, 2 species belonging to the genus *Metschnikowia* had a significantly different abundance. However, we were unable to resolve this further with certainty (S6 Table).

### Maximum temperature identified as a climatic factor driving the microbial communities of the 2021 vintage

**Fig 4:**
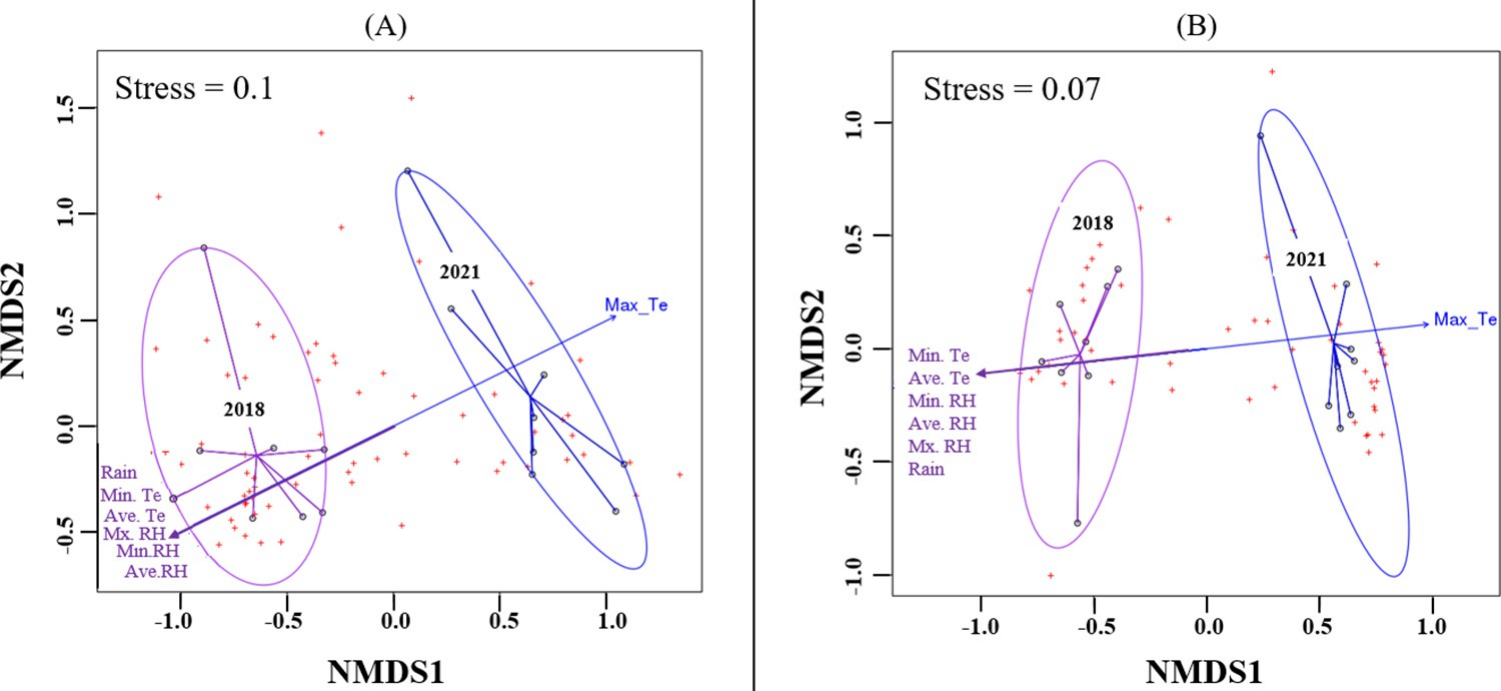
NMDS ordination for (A) Bacterial ASVs (B) Fungal ASVs associated with vintage 2018 and 2021 ferments. Fitting with environmental variables shows Maximum temperature (Max_Te) as the major factor influencing the 2021 vintage (blue) and the other climatic variables i.e Minimum temperature (Min. Te), Average temperature (Ave. Te), Minimum relative humidity (Min.RH), Average relative humidity (Ave. RH), Maximum relative humidity (Mx. RH), and rain, all correlating (S3 File) and having an impact on the 2018 community (purple). Red points depict the amplicon sequence variants (ASVs). Ellipses shown represent 95% confidence intervals.

NMDS plot fitted with environmental variables is shown in Fig 4. This plot shows clear demarcation based on vintage. Besides maximum temperature, which influenced the 2021 bacterial community, all other variables namely, minimum temperature (˚c), average temperature (˚c), minimum relative humidity (%), average relative humidity (%), maximum relative humidity (%), and rain (mm) were seen to influence the bacterial differences seen in the 2018 vintage (Fig 4A).

A similar pattern of segregation as seen in the bacterial community was observed for the fungal community. Clear separation occurred due to vintage with maximum temperature impacting the 2021 fungi community while the other variables correlated and were responsible for driving the 2018 fungi differences observed (Fig 4B).

## Discussion

The aim of this study was to examine the composition and stability of the bacterial and fungal communities associated with the spontaneous fermentation of Pinot noir wines over two vintages (2018 and 2021), and to consider if any fluctuations and changes observed could be related to climatic differences. Overall, vintage (and by inference, climate) appears to have more influence on bacterial diversity as compared to fungal diversity in this environment. The dominance and thus the importance of *Saccharomyces* irrespective of vintage is highlighted (Fig 3). In natural fermentation, the high abundance of *S*. *cerevisiae* has been attributed to the adaptations this organism has for proliferation in grape must [39]. This might suggest that indigenous *Saccharomyces* found on our grapes exerted and established dominance, especially in the last two stages of fermentation in both vintages. Furthermore, a significant compositional difference (log 2 fold change of 17.7; p.adj = 2.6e-^1^⁶) towards the 2018 vintage was reported (S6 Table). This possibly means that the species (or strains) of *Saccharomyces*, which dominate autochthonous fermentation, could vary from one vintage to another possibly due to climatic variations. Evidence for multiple strains of *S*. *cerevisiae* present in the 2018 vintage have been described before [19].

The non-*Saccharomyces* yeast detected in our study also displayed differences between the 2018 and the 2021 vintage (S5 Table). *Starmerella bacillaris* (*S*. *bacillaris*) was significantly different as our data indicated this was not detected in both replicates of vintage 2021 but was present in those of the 2018 vintage. The ability of *S*. *bacillaris* to survive during alcoholic fermentation has been reported [40–42]. Furthermore, it was recently identified as one of the yeasts associated with Tannat must of Bodega Garzón, a unique region in that the Atlantic Ocean bears influence on the nearby vineyards [41]. Organic treatments are known to have a positive influence on *S*. *bacillaris* i.e it increases their abundance [43]. Thus, since the same organic practices were applied in both vintages of our study, the different climatic conditions associated with the respective vintages may explain their absence in the 2021 vintage. Different research groups [44, 45] have previously outlined that *S*. *bacillaris* could stimulate an increase in the population of *Saccharomyces*, which might explain the significant compositional difference of *Saccharomyces* in vintage 2018.

Members of the *Hanseniaspora* genus favored the 2018 vintage (S5 Table). It has been reported that members of this genus are sensitive to ethanol concentration [46]. However, as with Lleixa, Manzano [47], our data shows that *Hanseniaspora* does persist till the end of fermentation. This observation was made in the 2018 and 2021 vintage, and previously where culture methods were used [19]. The enological importance of some species found in this genus has been established, namely *H*. *uvarum* [48], *H*. *vineae* [49, 50] and *H*. *guilliermondii* [51]. *Indeed*, it has been reported that *H*. *occidentalis* shows huge potential in reducing the malic acid content of wine made from Muscaris grape must [52]. For climatic variables, *Hanseniaspora* has previously shown a slightly positive correlation with rainfall and humidity [21, 53]. This is in line with our data which highlights both variables (R^2^ = 0.9633 –S3 File) as factors influencing the 2018 microbial diversity of which *Hanseniaspora* was found to be significantly abundant.

For vintage 2021, *Metschinikowia* and *Aureobasidium* were more bountiful in this vintage (S5 Table). Co-inoculation of some species of *Metschinikowia* with *S*. *cerevisiae*, has been used to produce wines with low alcohol content [54]. In enology, unwanted yeast genera such as *Penicillium* and *Aspergillus* are affected by the strong biocontrol activity of *M*. *pulcherrima* [55]. Milanović, Cardinali [56] recently reported the relative abundance of *Aureobasidium* in most samples to be inversely proportional with that of *Cladosporium*. Together, these might explain the limited abundance of saprophytic molds such as *Penicillium* observed in this vintage (S5 Table). Another reason might be directly linked to the climatic differences associated with both vintages. It has been established in the literature that relative humidity and rainfall favours fungal grapevine diseases [57]. Thus, this could explicate why the saprophytic molds mentioned above as well as other spoilage fungal organisms such as *Botryotinia*, were found to be differentially abundant in the 2018 vintage (S5 Table). Furthermore, the presence of *Aureobasidium* in our study is supported by other groups [58–60]. Their potential role in improving red wine during pre-fermentative maceration have also been highlighted [61, 62]. Most studies before now have associated *Aureobasidium* with the pre-fermentation period or freshly crushed grapes; our previous study of the 2018 vintage that used culture-based methods only detected strains in the earliest ferment stage [19]. For the precent study using culture-independent methods, its detection might be due to residual DNA from the high initial population. Additional research will be required to clarify this.

Other non-*Saccharomyces* yeast such as *Pichia kluyveri* and *Candida californica* occurred only in the 2018 vintage but with scanty abundance. *P*. *kluyveri* is the most studied among wine related *Pichia* species and is presently the only one available commercially [63]. Its role in influencing the sensory profile of wine has been reported [64–67]. Our result aligns with Wang, Hopfer [68] who showed that the genus *Pichia* was found in Chambourcin wine fermentations in one region but not in the other possibly due to climatic differences. *C*. *californica* has rarely been linked with Pinot noir fermentation musts. However, its presence has been detected in fermentation musts carried out with other grape varieties such as Isabella (*Vitis labrusca* L.) [69] and Grignolino grapes [70]. Together with other non-*Saccharomyces* yeast, it was shown to be capable of producing wines with less alcohol [71]. The presence of this genus in our result correlates with Zhang, Plowman [19] who used MALDI-TOF to characterize cultured yeast strains in Pinot noir musts from the same winery in the 2018 vintage.

In terms of bacteria, the most abundant genus found in the 2021 vintage was *Tatumella*. On the contrary, *Lactococcus* was the most dominant in the 2018 vintage, albeit with a lower abundance as compared to *Tatumella* (Fig 1 and S3 Table). Steenwerth, Morelan [21] have previously reported the dominance of *Tatumella* when working on Pinot noir must in the states of California and Oregon, USA. In our case, it is important to note that wines were spontaneously fermented and SO₂ was not added in both vintages. Thus, a high abundance of this genus was expected in both vintages since it has been established that its presence is severely impacted using SO₂ [72, 73]. However, with *Tatumella* abundance making up more than 50% of the 2021 bacteria community, climatic variations could be responsible for this difference. Specifically, maximum temperature as a variable associated with the 2021 vintage as indicated by our envfit model may be critical.

Similarly, the presence of *Lactococcus* in fermentation musts [74] and grape surfaces [75, 76] have been reported. *Lactococcus* is a member of the well-known lactic acid bacteria (LAB) family. Members of this family perform malolactic fermentation (MLF), which is significant in wine production. Other LAB which were differentially abundant include *Lactobacillus* (p.adj = 4.47e-^1^⁵) and *Fructobacillus* (p.adj = 7.6e-^1^⁹). *Lactobacillus* was relatively abundant in the 2018 vintage but limited in the 2021 vintage; *Fructobacillus* appeared absent from the 2018 vintage and present in the 2021 vintage (S3 and S4 Tables). Coupled with the stressors in a typical wine environment, this may indicate that *Lactobacillus* and *Fructobacillus* might have been impacted by climatic factors.

Acetic acid bacteria (AAB) such as *Komagataeibacter*, *Acetobacter*, and *Gluconobacter* appeared significantly abundant in the 2018 vintage (S3 and S4 Tables). Members of the AAB are responsible for the production of acetic acid and acetaldehyde and are thus associated with wine spoilage [77]. Our envfit model indicated rainfall and humidity as variables driving the 2018 vintage. This could possibly explain the high abundance of AAB observed in the 2018 vintage. Correspondingly, temperature plays a key role in the regulation of microbial growth optima with different microbial species benefiting differently [78]. In our study, Fig 4 highlight this, with maximum temperature influencing both the bacterial and fungal community of the 2021 vintage. In addition, though the amount of rain associated with both vintages was not significantly different, the higher range (1 mm in November of 2017 versus 185 mm in February of 2018) found in the 2018 vintage might be a factor contributing to the higher microbial diversity associated with this vintage. This is in line with Steenwerth, Morelan [21] who concluded that growing season precipitation shapes bacterial beta diversity. Further studies will be needed to affirm this. Nonetheless, our study and those others aforementioned strongly indicate that changes in climatic conditions may influence the microbiome of wine fermentation musts. These microbial differences could then possibly have an impact on chemical and aroma composition of the final wine product as previously described [79, 80].

## Conclusion

Variations were reported by Zhang, Plowman [19] in the species and intraspecific communities of yeast cultured from winemaking fermentation tanks used to produce organic Pinot noir wine, when MALDI-TOF was utilized to characterize cultured yeast strains in 2018. Results from the aforementioned study align with that of Ohwofasa, Dhami [20] when metabarcoding approaches were utilized to monitor the bacterial and fungal populations in the winemaking process across the entire alcoholic fermentation period. Interestingly, several non-*Saccharomyces* yeasts reported in the former study were not found in the latter. This pointed to the likelihood that due to climatic variations, microbial diversity might differ from year to year even within a sub region. We followed up with this study and compared the microbial diversity of corresponding time points across two vintages using a culture-independent, metabarcoding approach with biological replicates from the same wine production facilities.

For bacteria, we show that the starting diversity associated with the 2018 vintage was dominated by *Lactococcus* while the 2021 vintage was dominated by *Tatumella*. Our fungal data revealed that, besides *Saccharomyces*, *Starmerella* was copious in the 2018 vintage while *Metschnikowia* was in the 2021 vintage. It is noteworthy to mention that fermentation in both vintages relied on an autochthonous fermentation style with no SO₂ added. Therefore, any microbial differences may likely be due to differing factors, of which climate has been indicated to be one such feature. Finally, we acknowledge that using metabarcoding to uncover the presence of microorganisms does not always mean they are active. Thus, culture-based approaches might be required to compliment this study in the future. Nevertheless, we believe this study presents valuable information to winemakers by emphasizing the impact climatic variation might exert on microbial communities associated with wine fermentation processes. Such variations may have deep and long-lasting relevance on production methods and quality control procedures for winemakers facing, as we all do, the challenges driven by climate change.

## Supporting information

S1 Fig Climatic variables observed in both vintages. (PDF)

S2 Fig Fungal alpha diversity. Fungal alpha diversity (Shannon diversity index) of the ferment samples from (A) Replicate A; (B) Replicate B. No significant difference (p > 0.05) were observed. The same conclusion can be drawn from both replicates.
(TIF)

S3 Fig Beta Diversity using PERMANOVA for (A) Bacteria (B) Fungi communities.
(TIF)

S1 File Code used for fungal data analysis. (TXT)

S2 File Code used for bacterial data analysis. (TXT)

S3 File Envfit output after fitting ASVs with climatic data. (TXT)

S1 Table Averaged climatic data used as environmental variable. (XLSX)

S2 Table Temperature observations. (XLSX)

S3 Table Bacterial genera count in both vintages. (CSV)

S4 Table DESeq2 output showing significantly differentially expressed bacterial ASVs. (CSV)

S5 Table Fungal genera count in both vintages. (CSV)

S6 Table DESeq2 output showing significantly differentially expressed fungal ASVs. (CSV)

S7 Table Sequencing statistics. (CSV)
