# Supplementary material for: Influence of climatic variation on microbial communities during organic Pinot noir wine production
Source: PLoS One. 2024 Feb 28;19(2):e0296859. doi: 10.1371/journal.pone.0296859 (PMC10901304; doi:10.1371/journal.pone.0296859)
Supplement: S1 Fig — (PDF) [file pone.0296859.s001.pdf]

(A)

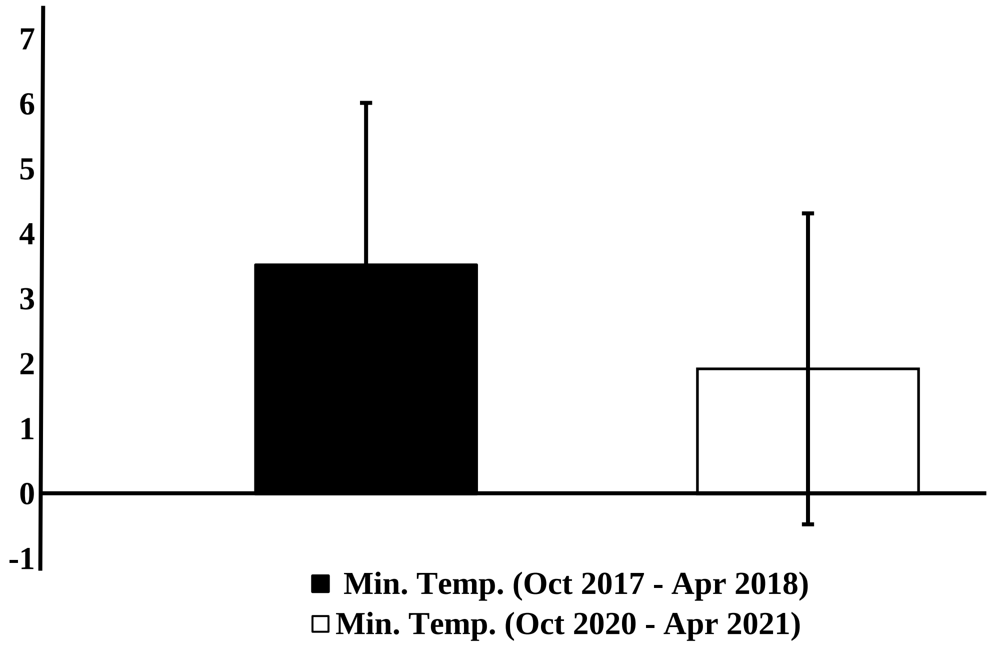

S1 FigA: Minimum temperature (Average ± S.D) observed in both vintages. No significant difference was observed ($p > .05$)

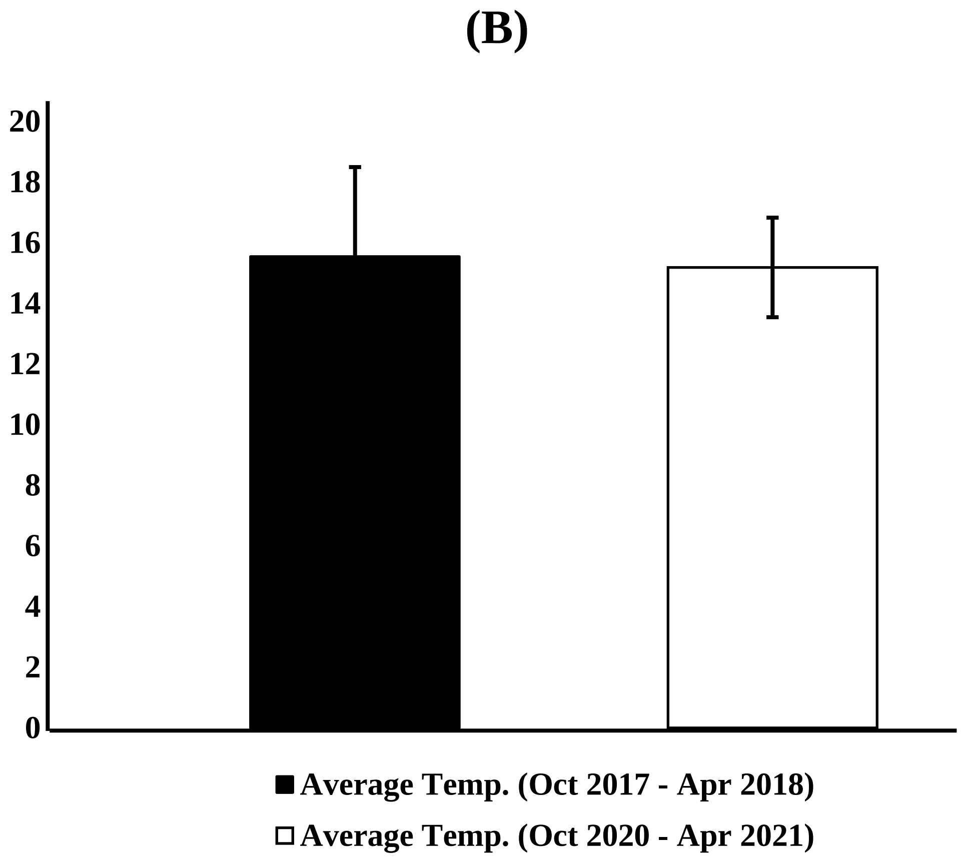

S1 FigB: Average temperature (Average ± S.D) observed in both vintages. No significant difference was observed (p > .05)

(C)

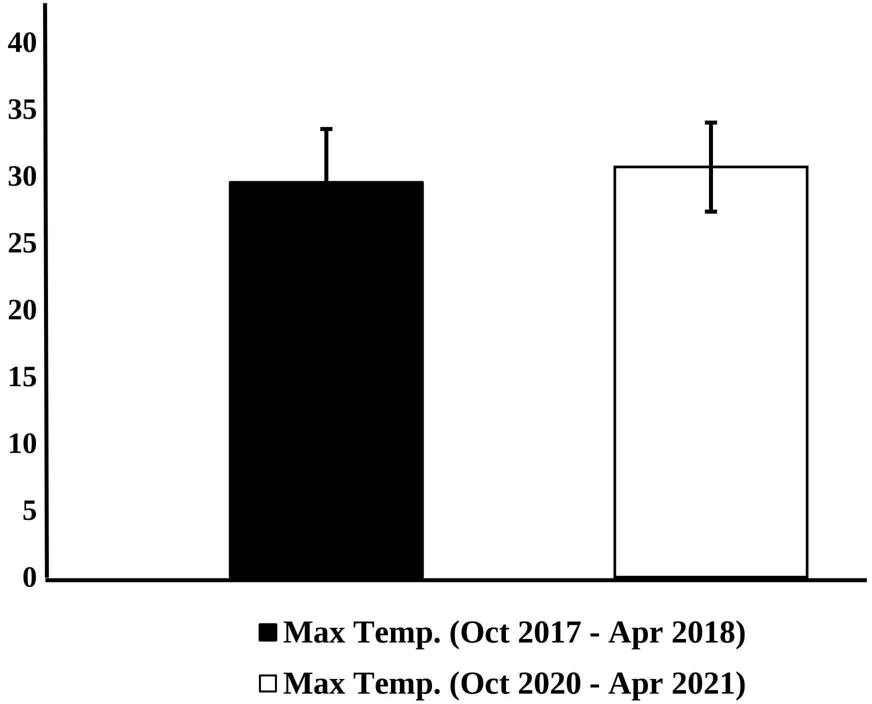

S1 FigC: Maximum temperature (Average ± S.D) observed in both vintages. No significant difference was reported (p > .05)

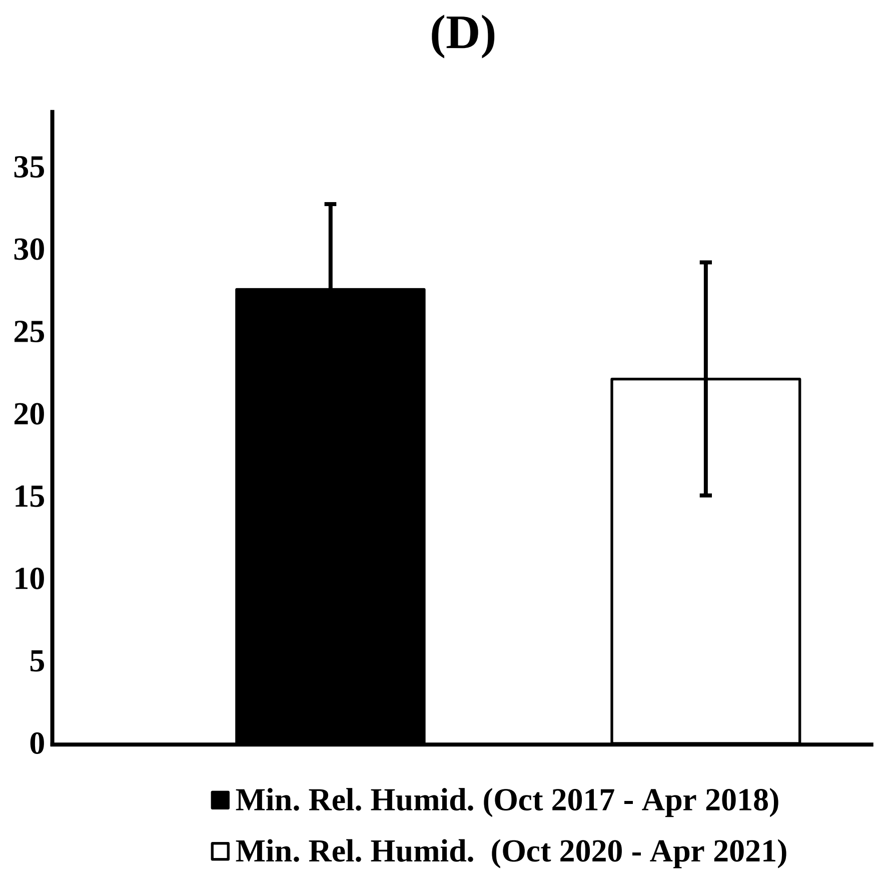

S1 FigD: Minimum relative humidity (Average ± S.D) observed in both vintages. No significant difference was observed (p > .05)

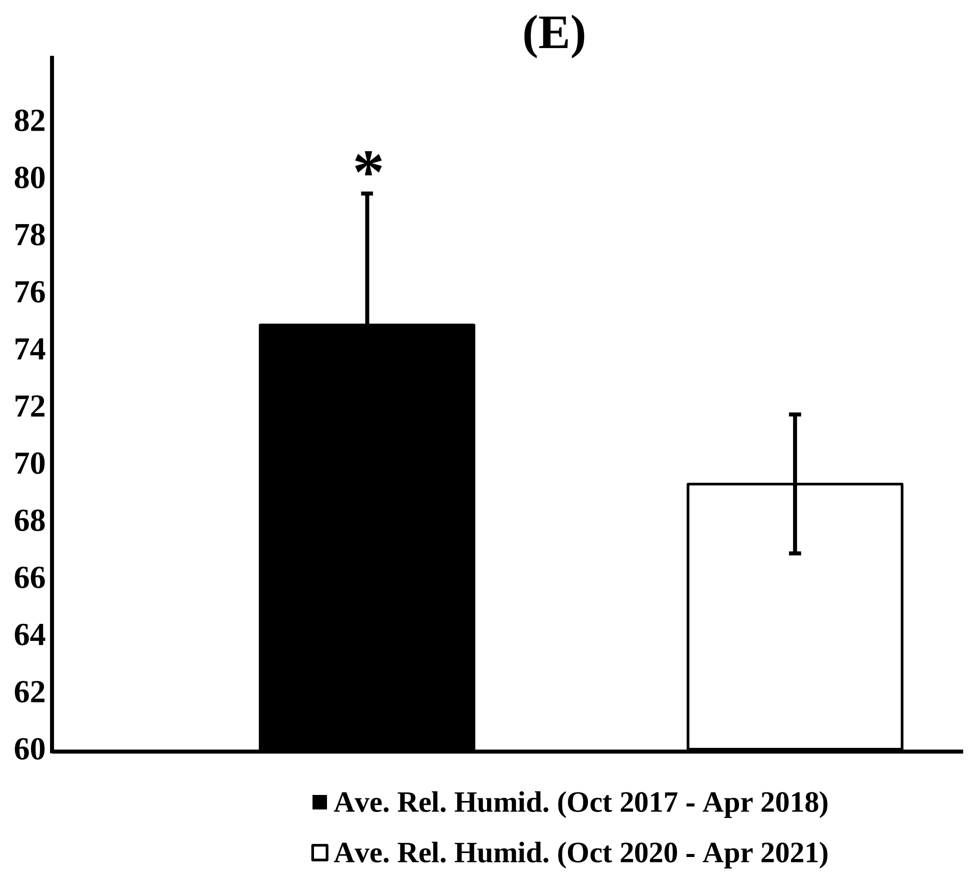

S1 FigE: Average relative humidity (Average ± S.D) observed in both vintages. Significant difference observed (p = .02503)

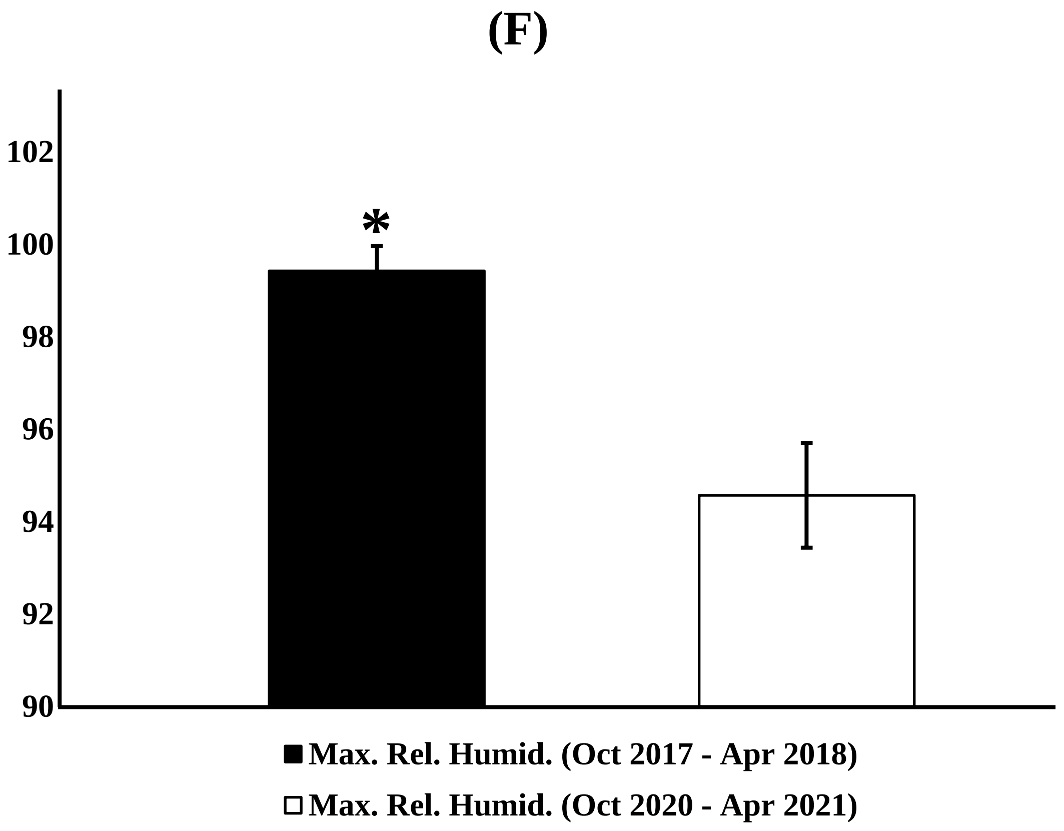

S1 FigF: Maximum relative humidity (Average ± S.D). Significant difference observed (*p* = .001732)

(G)

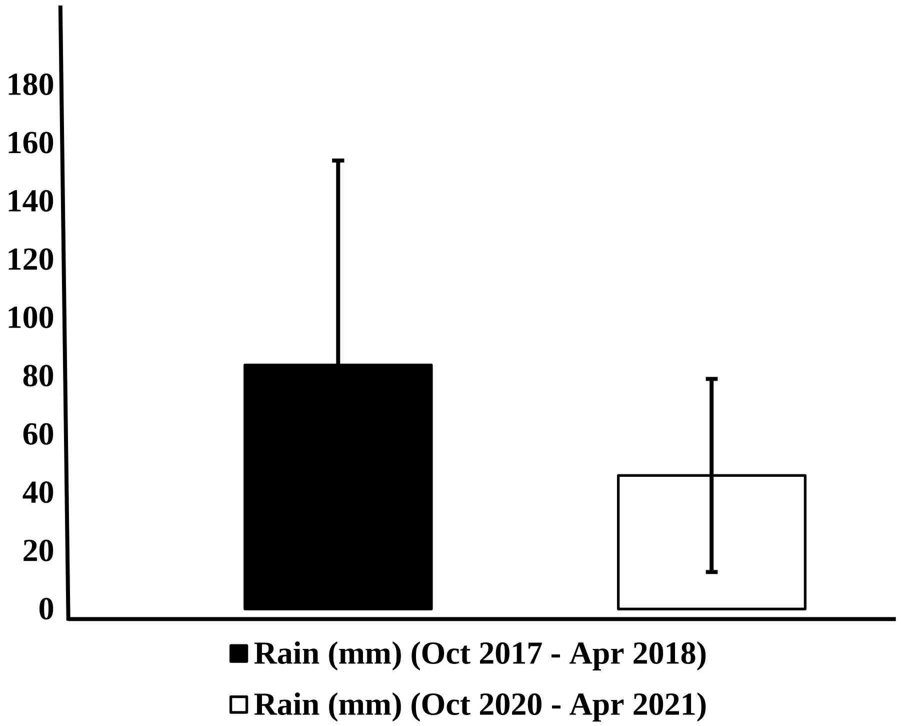

S1 FigG: Rain (Average ± S.D) observed in both vintages. No significant difference was reported ($p > .05$)
