# Supplementary figures and images for: Influence of climatic variation on microbial communities during organic Pinot noir wine production
Source: PLoS One. 2024 Feb 28;19(2):e0296859. doi: 10.1371/journal.pone.0296859 (PMC10901304; doi:10.1371/journal.pone.0296859)

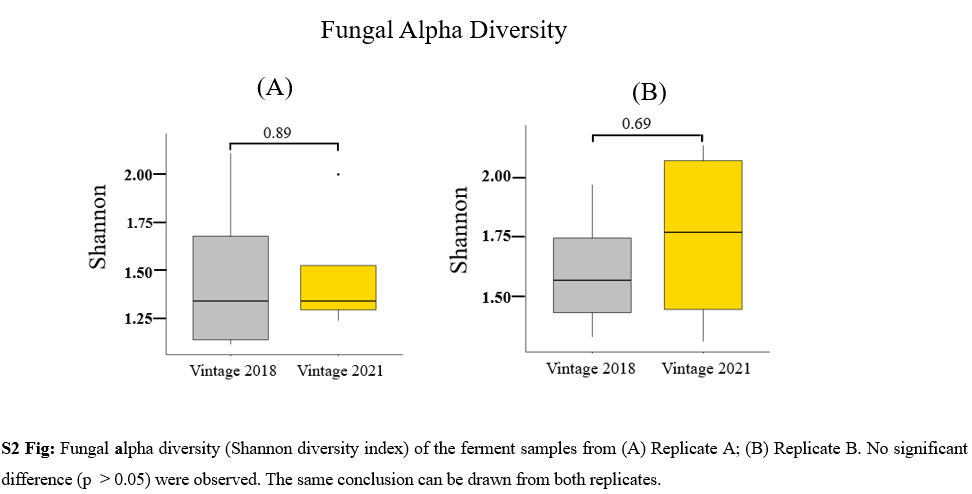

Supplement: S2 Fig — Fungal alpha diversity (Shannon diversity index) of the ferment samples from (A) Replicate A; (B) Replicate B. No significant difference (p > 0.05) were observed. The same conclusion can be drawn from both replicates. (TIF) [file pone.0296859.s002.tif]

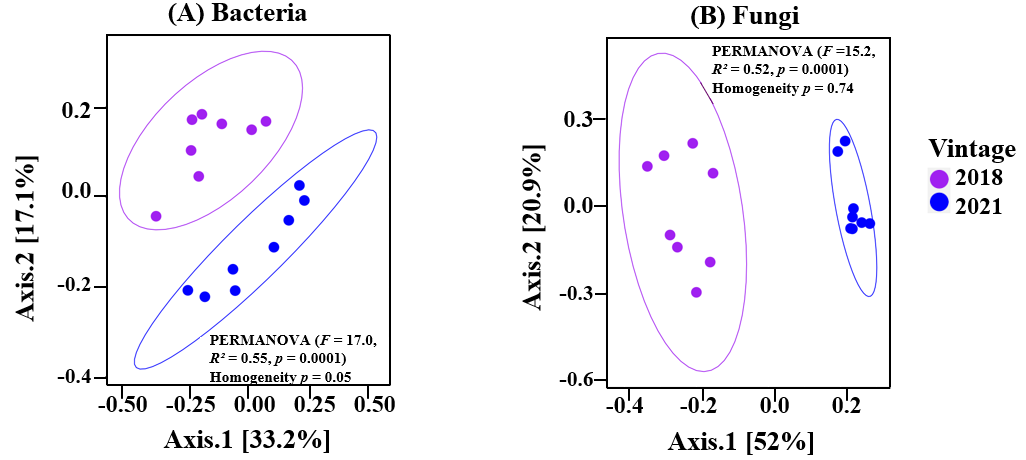

Supplement: S3 Fig — Beta Diversity using PERMANOVA for (A) Bacteria (B) Fungi communities. (TIF) [file pone.0296859.s003.tif]
